# Organization of the parallel antennal-lobe tracts in the moth

**DOI:** 10.1007/s00359-022-01566-x

**Published:** 2022-09-16

**Authors:** Jonas Hansen Kymre, Xi Chu, Elena Ian, Bente Gunnveig Berg

**Affiliations:** Chemosensory Lab, Department of Psychology, Norwegian University of Science and Technology, Trondheim, Norway

**Keywords:** Insect olfaction, Antennal-lobe tracts, Projection neurons, Neuroanatomy, Electrophysiology

## Abstract

The olfactory pathways of the insect brain have been studied comprehensively for more than 40 years, yet the last decade has included a particularly large accumulation of new information relating to this system’s structure. In moths, sharp intracellular recording and staining has been used to elucidate the anatomy and physiology of output neurons from the primary olfactory center, the antennal lobe. This review concentrates on the connection patterns characterizing these projection neurons, which follow six separate antennal-lobe tracts. In addition to highlighting the connections between functionally distinct glomerular clusters and higher-order olfactory neuropils, we discuss how parallel tracts in the male convey distinct features of the social signals released by conspecific and heterospecific females. Finally, we consider the current state of knowledge regarding olfactory processing in the moth’s protocerebrum and make suggestions as to how the information concerning antennal-lobe output may be used to design future studies.

## Introduction

Insects rely on their outstanding chemical sense to detect biologically relevant odor cues transforming the associated neuronal signals into essential behavioral outcomes, such as foraging, mate searching, and selecting sites for oviposition. Each of these complex behaviors are regulated by circuits constituted by considerably fewer neurons than those forming corresponding networks in mammals (Mizunami et al. 2004). Studying the chemosensory circuits in insects, which are involved in several stereotypical innate behaviors (e.g., Karlson and Butenandt 1959), is ideal for understanding how neuroarchitecture defines circuit function. In this review, we endeavor to inspect the organization of the output neurons from the moth`s primary olfactory center, the antennal lobe (AL), which receive input from local interneurons and olfactory sensory neurons situated on the antennae.

**Fig. 1:**
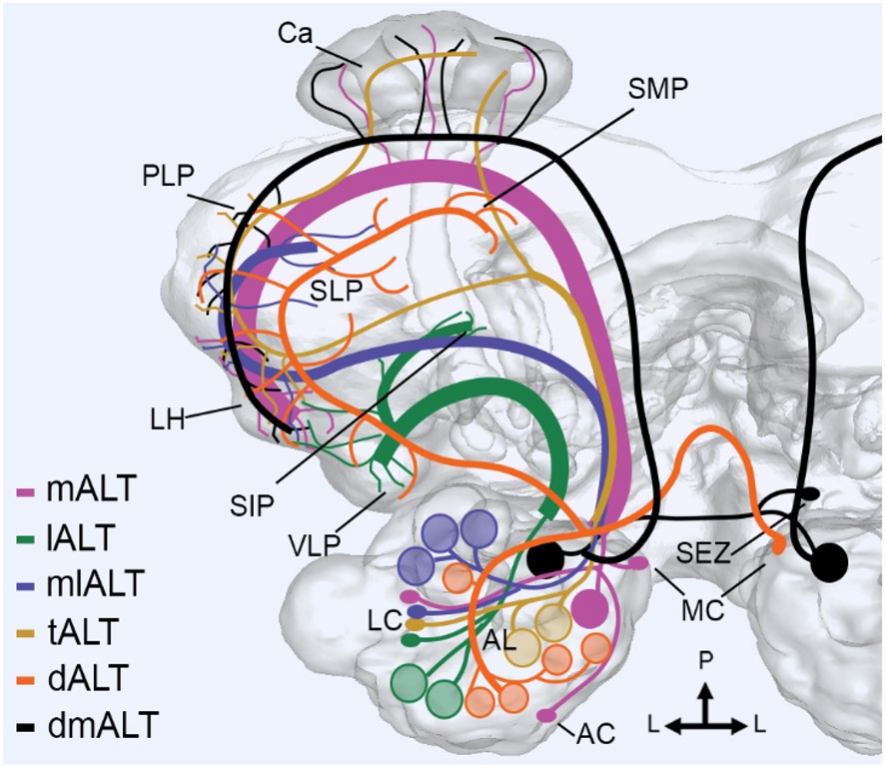
Schematic overview of the six antennal-lobe tracts (ALTs) in Lepidoptera. While the medial and dorsomedial tracts (mALT and dmALT, respectively) predominantly have uniglomerular projection neurons (PNs), the remaining tracts mainly have PNs with some extent of multiglomerular dendrites, as represented by the differing number of circles in the antennal lobe (AL). The six ALTs follow separate pathways to several protocerebral target regions, of which the lateral horn (LH) is the most heavily innervated. Note that the neuropils marked in the figure is by no means exhaustive—for more details see Table 1. *AC*, anterior cell-body cluster; *Ca*, calyces of the mushroom body; *dALT*; dorsal ALT; *lALT*, lateral ALT; *LC*, lateral cell-body cluster; *MC*, medial cell-body cluster; *mlALT*, mediolateral ALT; *PLP*, posteriorlateral protocerebrum; *SEZ*, subesophageal zone; *SIP*, superior intermediate protocerebrum; *SLP*, superior lateral protocerebrum; *SMP*, superior medial protocerebrum; *tALT*, transverse ALT; *VLP*, ventrolateral protocerebrum. Figure copied from Kymre et al. (2021a)

Like mammals, insects possess a direct connection between the primary olfactory center and higher brain centers, including regions for memory establishment and odor-evoked innate behaviors (reviewed in Chakraborty and Sachse 2021; Knaden and Hansson 2014). This connection is provided by uniglomerular output neurons forming the olfactory tract and medial antennal-lobe tract (mALT) in mammals and insects, respectively. However, insects also possess a large variety of output neurons with multiglomerular dendrites. The insect`s AL output neurons, termed projection neurons (PNs), follow several parallel tracts which are associated with distinct but partially comparable dendritic sampling strategies across orders (Galizia and Rössler 2010). In moths, there are a total of six tracts in each hemisphere (Fig. 1; see Table 1 for overview of all reported PN sub-types)—three main tracts, named the medial, lateral, and mediolateral ALT (mALT, lALT, and mlALT, respectively), and three minor tracts called the transverse, dorsomedial, and dorsal ALT (tALT, dmALT, and dALT, respectively; Homberg et al. 1988; Ian et al. 2016a; Namiki and Kanzaki 2011). The anatomical organization of these tracts was thoroughly described by Homberg et al. (1988) in a study on the sphinx moth, *Manduca sexta*. The review presented here builds on this previous publication and subsequent investigations (see Table 1 for overview of relevant literature)—in particular, articles showing high-resolution confocal data of separately labeled neurons. Recently, more than 100 individual antennal-lobe PNs were morphologically characterized and displayed in a noctuid moth, *Helicoverpa armigera*, each of which was confined to one of the six ALTs (Kymre et al. 2021a). Example confocal images and 3D reconstructions of distinct PN sub-types are available via the insect brain database (https://insectbraindb.org/app/neuron/dataset/5fa25351-9cc8-4796-beed-74e13e7dae02). In addition, PNs innervating specific sub-sets of glomeruli processing input about pheromones and CO_2_ have also received substantial attention lately (Chu et al. 2020a, b; Kymre et al. 2021b). Altogether, the data show that tracts other than the mALT are formed mainly by axons of multiglomerular PNs, except for the dmALT consisting primarily of bilaterally uniglomerular PNs. Together with former findings, the set of new anatomical data containing many novel PN types contributes to an increased understanding of the role of each separate olfactory tract, especially in terms of how these tracts represent specific glomerular clusters.

**Table 1:** Previously reported PN morphologies in Lepidoptera

ALT	Sub-type	FKA	AL innerv	Non-AL innerv	Soma	References
**mALT**	Pm_a	PIa	**UG**; MG	Ca; LH (SLP; SIP)	A-, L-, MC	[1, 4–10, 12–16, 18–19]
	Pm_b	Pm_d	UG	Ca; LH; VLP; INP	LC	[6]
	Pm_c	Pm_e	UG; **MG**	SEZ; AMMC; SLP; ICL; PLP; LH; VLP; bCa	AC	[2, 6, 9]
	Pm_d	Pm_f	MG	CL. VMNP; PLP	LC	[6]
	Pm_e	Pm_g	UG	CL. LH	LC	[3]
**lALT**	Pl_a_uni	POa	MG	AL Isthmus; Col	LC	[3, 4–6, 10–11]
	Pl_a_bi	−	UG; MG	Col.; LH; PED; CL. Col	LC	[3, 6, 11, 17]
	Pl_b	POb	UG; MG	VLP	LC	[5–6, 9, 15]
	Pl_c	POc	UG; MG	LH; PLP; Ca	LC	[4–5, 9, 13, 15]
	Pl_d	POd	UG; MG	AMMC; SEZ; VLP; TC; LH	LC	[5, 9]
	Pl_e	−	MG	VLP; SLP	LC	[6, 9]
	Pl_f	−	MG	LH; INP	LC	[6]
	Pl_g	−	UG	LH, SLP, PLP	LC	[2]
**mlALT**	Pml_a	PM	MG	LH; (VLP; SLP)	LC	[5–6, 10–11, 15]
	Pml_b	PM	UG; **MG**	LH; SLP; SMP; bCa	LC	[5–6, 9–10, 13, 15]
	Pml_c	−	UG; **MG**	LH, PLP; SLP, ICL, bCa	LC	[9]
	Pml_d	PM	MG	VLP; LH; SLP; SIP; SMP; CRE	LC	[5, 9, 15]
**tALT**	Pt_a	PIc	UG; **MG**	LH; PLP; SLP; Ca	LC	[2, 5, 9, 15]
	Pt_b	−	UG	SEZ; AMMC; BL. Ca; PLP; SLP	LC	[9]
	Pt_c	−	MG	SLP; PLP	LC	[9]
	Pt_d	PIb	UG; MG	SMP; SLP, PLP	LC	[2, 5, 9]
	Pt_e	−	BL. UG	CL. LH; PLP	MC	[2]
	Pt_f	−	UG	LH; BL. VLP	LC	[2]
	Pt_g	Pt	-	INP; LH; VLP; bCa; SEZ	LC	[6]
**dmALT**	Pdm_bi	−	BL. UG	BL. Ca; LH; PLP	GCBR	[6–7, 9, 13, 15]
	Pdm_uni	−	UG	SMP, INP, VMNP, AMMC, SEZ	-	[2, 15]
**dALT**	Pd	−	MG	CL. VLP; PLP; LH; bCa; SLP; SIP; SMP	CL. MC	[5, 7, 9, 13]

Bold indicates dendritic innervations occurring most frequently. Parentheses indicate projection patterns associated with MGC PNs. Table adapted from Kymre et al. (2021a)

References: (1) Anton et al. (1997); (2) Chu et al. (2020b); (3) Chu et al. (2020a); (4) Hansson et al. (1994); (5) Homberg et al. (1988); (6) Ian et al. (2016b); (7) Kanzaki et al. (1989); (8) Kanzaki et al. (2003); (9) Kymre et al. (2021a); (10) Kymre et al. (2021b); (11) Lee et al. (2019); (12) Løfaldli et al. (2010); (13) Namiki and Kanzaki (2011); (14) Nirazawa et al. (2017); (15) Rø et al. (2007); (16) Sadek et al. (2002); (17) Wu et al. (1996); (18) Zhao and Berg (2010); (19) Zhao et al. (2014)

*AC*, anterior cell cluster; *AL*, antennal lobe; *ALT*, antennal-lobe tract; *AMMC*, antennal mechanosensory and motor center; *BL*, bilateral; *Ca*, calyces; *CL*, contralateral; *Col*, column of the superior intermediate protocerebrum; *dALT*, dorsal ALT; *dmALT*, dorsomedial ALT; *FKA*, formerly known as; *GCBR*, cell-body rind around the gnathal ganglion; *INP*, inferior neuropil; *lALT*, lateral ALT; *LC*, lateral cell cluster; *LH*, lateral horn; *mALT*, medial ALT; *MC*, medial cell cluster; *MG*, multiglomerular; *mlALT*, mediolateral ALT; *PED*, peduncle of the mushroom body; *PLP*, posteriorlateral protocerebrum; *SEZ*, subesophageal zone; *SLP*, superior lateral protocerebrum; *SNP*, superior neuropil; *tALT*, transverse ALT; *TC*, tritocerebrum; *UL*., unilateral; *UG*, uniglomerular; *VLP*, ventrolateral protocerebrum; *VMNP*, ventromedial neuropil

## Organization of the antennal-lobe glomeruli

Built upon previous (Berg et al. 2002; Greiner et al. 2004; Kazawa et al. 2009; Rospars & Hildebrand 1992; Skiri et al. 2005) and newer findings (Zhao et al. 2016), the AL glomeruli of moths are principally divided into four clusters with putative functional differences: (i) The dorsolaterally located sex-specific complex—termed the macroglomerular complex (MGC) in males and the female-specific complex (Fx) in females, with the processing of female-released pheromones in the MGC being particularly well studied (Berg et al. 1998; Christensen & Hildebrand 1987; Hansson et al. 1994; Kanzaki et al. 2003; Vickers et al. 1998; Zhao et al. 2014); (ii) the dorsoposteriorly located posterior complex (PCx) which has not yet been functionally characterized; (iii) the general-odorant processing ordinary glomeruli (OGs), found throughout medial, anterior, dorsal, and some ventral parts of the AL; and (iv) the CO_2_-responsive labial palp-pit organ glomerulus (LPOG; Chu et al. 2020b; Guerenstein et al. 2004; Kent et al. 1986). In addition, across-species morphological similarities of both glomerular arrangement and PN structure recently led us to propose (Kymre et al. 2021a) that a group of ventroposterior glomeruli (VPGs) may serve a function corresponding to that found in other insect orders, i.e., processing information about temperature and humidity (Enjin et al. 2016; Frank et al. 2015, 2017; Marin et al. 2020; Nishino et al. 2003). The function of the VPGs has not yet been experimentally validated in any lepidopteran species.

## The three main ALTs: the medial, lateral, and mediolateral tract

Data obtained from mass-staining experiments of various AL regions consistently include the three main ALTs (Homberg et al. 1988; Ian et al. 2016a; Rø et al. 2007; Seki et al. 2005). Each of these paths thereby appears to connect with all glomerular clusters in the moth AL. Indeed, individual PNs with omniglomerular innervations are found in both the lateral and mediolateral tracts, while the mainly uniglomerular medial-tract PNs collectively cover all glomerular sub-groups (Chu et al. 2020b; Kymre et al. 2021a, b).

### The medial ALT

As in other insects, a prominent fiber bundle of uniglomerular PN axons constitutes the mALT of moths (Hansson et al. 1994; Homberg et al. 1988; Ian et al. 2016b; Kanzaki et al. 1989; Namiki and Kanzaki 2011). Owing to their limited dendritic arborization, the relatively homogenous population of mALT neurons are assumed to be essential for odor identification and discrimination. Like the mammalian mitral and tufted cells, they connect each individual glomerulus in the primary processing center with target areas in the higher brain centers – a projection pattern optimally suited for fine-tuned odor coding including odor-associative memory based on experience. Notably, the neuropil specifically devoted to memory formation in insects, the mushroom body calyces (reviewed in Heisenberg 2003), is substantially innervated by the uniglomerular mALT PNs; while the PNs of the other tracts, being mainly multiglomerular, usually refrain from targeting this structure (Homberg et al. 1988; Ian et al. 2016b; Kymre et al. 2021a).

For the medial-tract PNs, there is a marked change in innervation pattern at the level of the protocerebrum as compared to the AL. Specifically, the conspicuous pattern of spatially restricted uniglomerular dendritic innervation is exchanged with widespread and partially overlapping terminal projections in protocerebral neuropils such as the lateral horn (LH) and calyces. The seemingly unorganized protocerebral branches include a form of map, however, as there is a spatial segregation of medial-tract projections conveying pheromone versus plant odor information (Chu et al. 2020a; Homberg et al. 1988; Namiki et al. 2013; Seki et al. 2005; Zhao et al. 2014). This pattern of separated projections applies both to the memory center, i.e., the calyces, and centers associated with innate behavior in the lateral protocerebrum, where similar patterns have been reported in the fruit-fly, *Drosophila melanogaster* (Jefferis et al. 2007). In addition, we recently demonstrated segregated processing of distinct social cues conveyed along the medial tract of *H. armigera* males. Specifically, mALT PNs responding to the primary pheromone component predominantly innervates the superior lateral protocerebrum (SLP) and superior intermediate protocerebrum (SIP), whereas mALT PNs sensitive to interspecific signals and the secondary pheromone component (acting as a behavioral antagonist at high concentration) rather innervate anterior parts of the LH (Kymre et al. 2021b). In the silkmoth, *Bombyx mori*, Seki et al. (2005) demonstrated that mALT PNs responding to the major pheromone terminate medially of neurons tuned to the minor-pheromone. However, they reported that terminals of these PNs overlap within lateral parts of the region referred to as the delta region of the lateral protocerebrum. In our recent work, which followed the standardized anatomical nomenclature introduced by Ito et al. (2014), we also found that all medial-tract MGC PNs have a common output target, i.e., anterodorsal parts of the ventrolateral protocerebrum (VLP). Yet, as mentioned previously, the functionally distinct PNs, associated with attractive and aversive female-produced odorants, had additional terminals in non-overlapping neuropils (Kymre et al. 2021b).

**Fig. 2:**
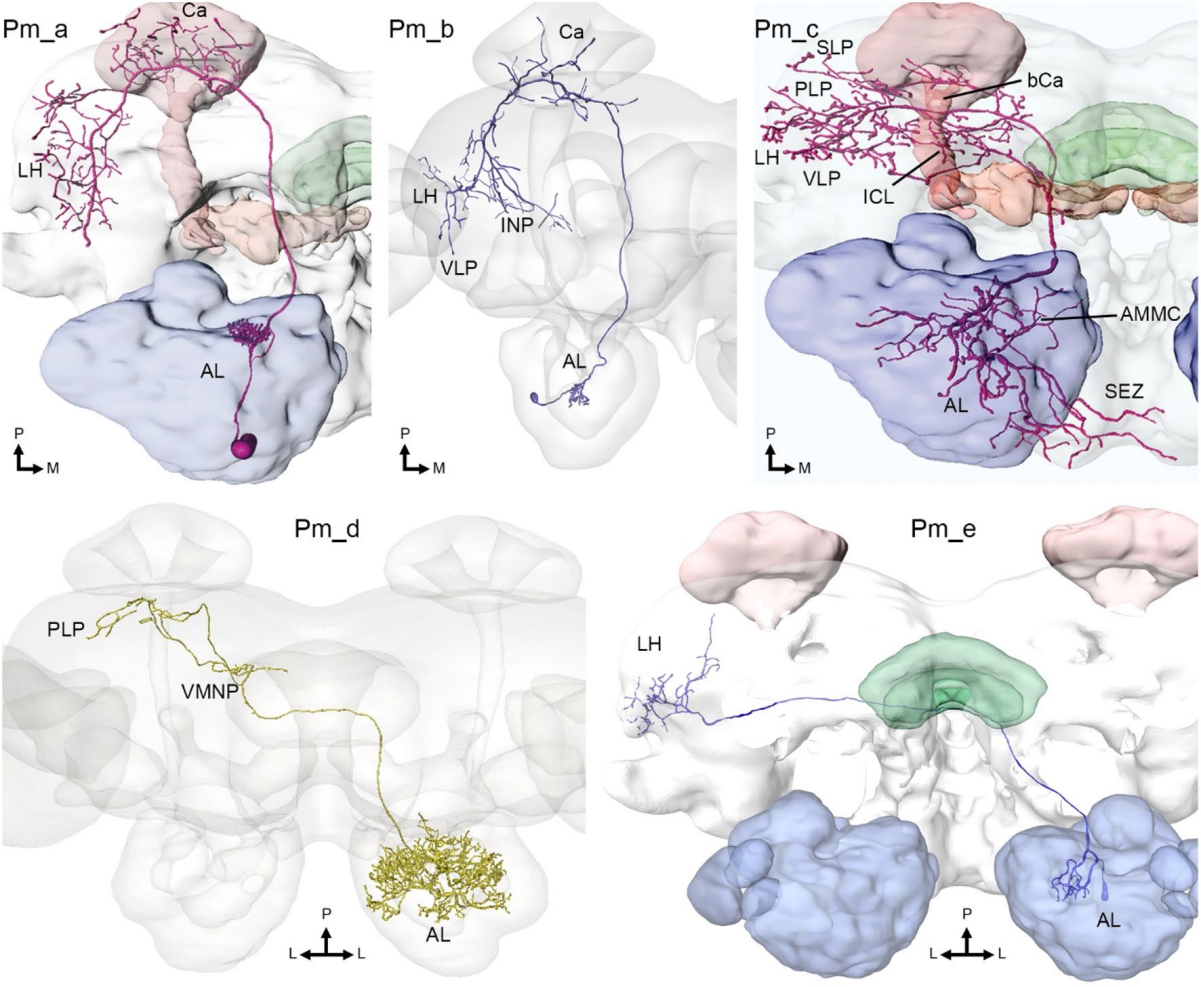
Reported medial-tract PN sub-types in Lepidoptera. *AL*, antennal lobe; *bCa*, base of the calyces of the mushroom body; *Ca*, calyces; *ICL*, inferior clamp; *INP*, inferior neuropils; *LH*, lateral horn; *PLP*, posteriorlateral protocerebrum; *SLP*, superior lateral protocerebrum; *VLP*, ventrolateral protocerebrum; *VMNP*, ventromedial neuropils. Adapted from Ian et al. (2016b), Kymre et al. (2021a), and Chu et al. (2020b)

In addition to the well-known uniglomerular PNs described above, called Pm_a, which constitutes the majority of the ca. 400 mALT axons (estimates from *M. sexta* males, Homberg et al. 1988), there are five other reported mALT sub-types. Here, it should be noted that the PNs previously known as Pm_b and Pm_c were recently found to project through the transverse tract, and thus recategorized as Pt_d and Pt_a, respectively (Kymre et al. 2021a). Consequently, we propose an update to the terminology of the medial-tract PNs to fill the void left by moving two PN sub-types from the medial to the transverse tract (see Table 1). Amongst the most notable mALT PN sub-types, is the Pm_c sub-type (previously referred to as Pm_e; Fig. 2). Contrary to most mALT PNs, these neurons are mainly oligoglomerular and terminate in extensive areas of the ipsilateral protocerebrum, but not in the calycal cups (Ian et al. 2016b; Kymre et al. 2021a). They also have large somata located in the anterior cell-body cluster, which comprise only ~ 16 somata, and some extend dendrite-like arbors in the subesophageal zone, the antennal mechanosensory and motor center, and ventral AL glomeruli, including the LPOG or VPGs (Chu et al. 2020b; Kymre et al. 2021a). These neurons may thereby integrate inputs across several distinct sensory modalities.

### The lateral ALT

**Fig. 3:**
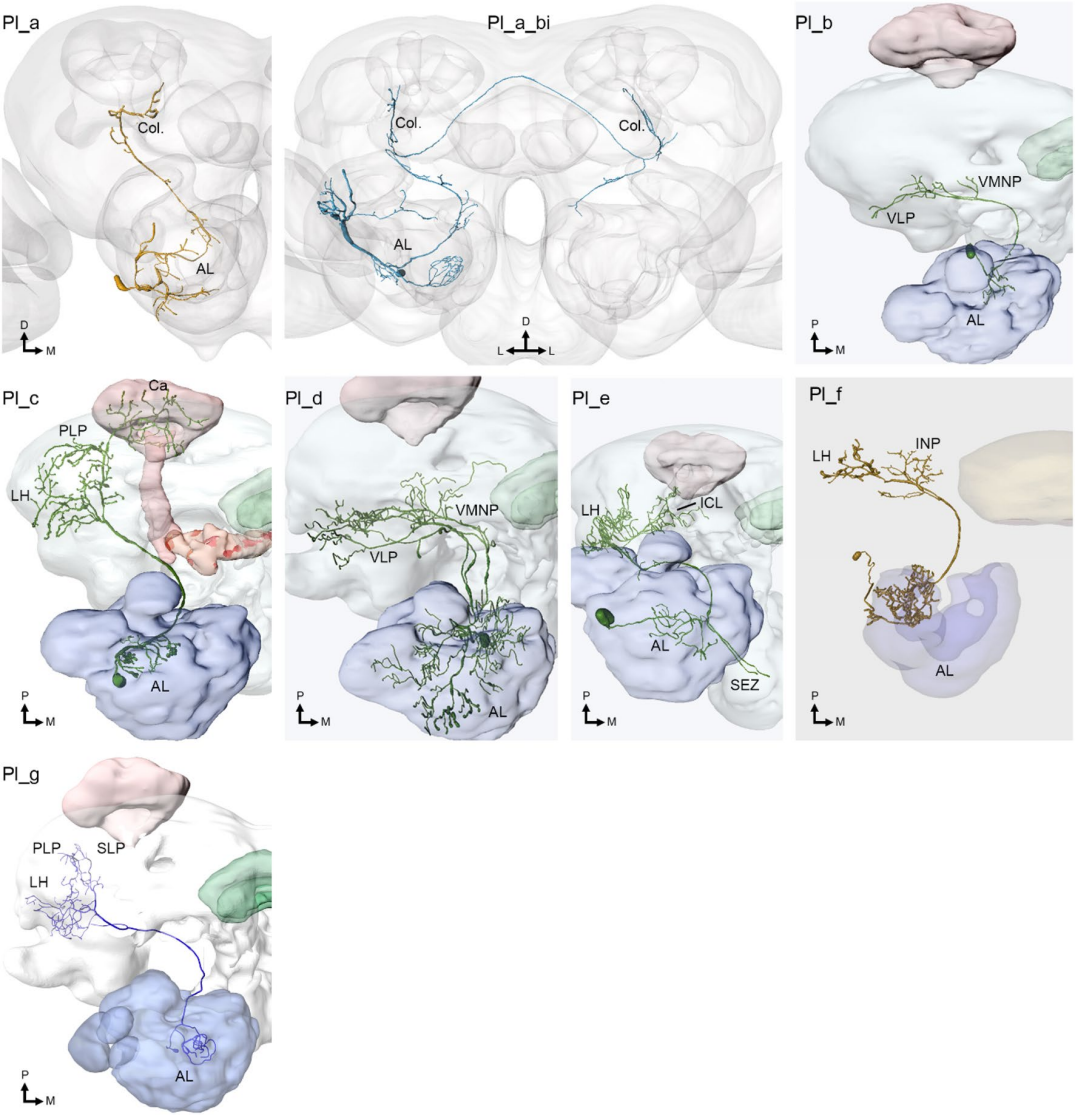
Reported lateral-tract PN sub-types in Lepidoptera. *AL*, antennal lobe; *Ca*, calyces of the mushroom body; *Col.*, column of the superior intermediate protocerebrum; *ICL*, inferior clamp; *INP*, inferior neuropils; *LH*, lateral horn; *PLP*, posteriorlateral protocerebrum; *SEZ*, subesophageal zone; *SLP*, superior lateral protocerebrum; *VLP*, ventrolateral protocerebrum; *VMNP*, ventromedial neuropils. Adapted from Ian et al. (2016b), Kymre et al. (2021a), and Chu et al. (2020b)

By being constituted of approximately 350 PN axons (Homberg et al. 1988), the lALT (Fig. 3) is the second most numerous ALT in moths. Unlike the mainly homogenous mALT PNs described above, those in the lateral tract include enormously heterogeneous morphological sub-types projecting to different output regions in the protocerebrum. One sub-type, i.e., the Pl_a neurons, is especially interesting because this sub-group has dendritic innervation of either ordinary glomeruli or the MGC, yet all its terminals converge onto the column of the SIP (Chu et al. 2020a; Ian et al. 2016a; Lee et al. 2019). It is worth noting that the column appears to be the only output area in the moth brain receiving a substantial number of overlapping terminals from PNs devoted to both the plant odor and pheromone system. Opposite from the mALT, which segregates the PN terminals of the two AL sub-systems, this branch of the lALT integrates them within a strikingly delimited area. Homberg et al. (1988) identified this sub-type of lALT PNs in *M. sexta*. They reported a numerous neuronal sub-category including both uniglomerular MGC PNs and multiglomerular PNs connected with the ordinary glomeruli, both terminating within a narrow area between the anterior optic tubercle and the vertical lobe, identical to the column. These findings, which seem to have been partly overlooked for a long period, are in full correspondence with subsequent data from heliothine moths (Chu et al. 2020a; Ian et al. 2016b; Lee et al. 2019; Rø et al. 2007).

**Fig. 4:**
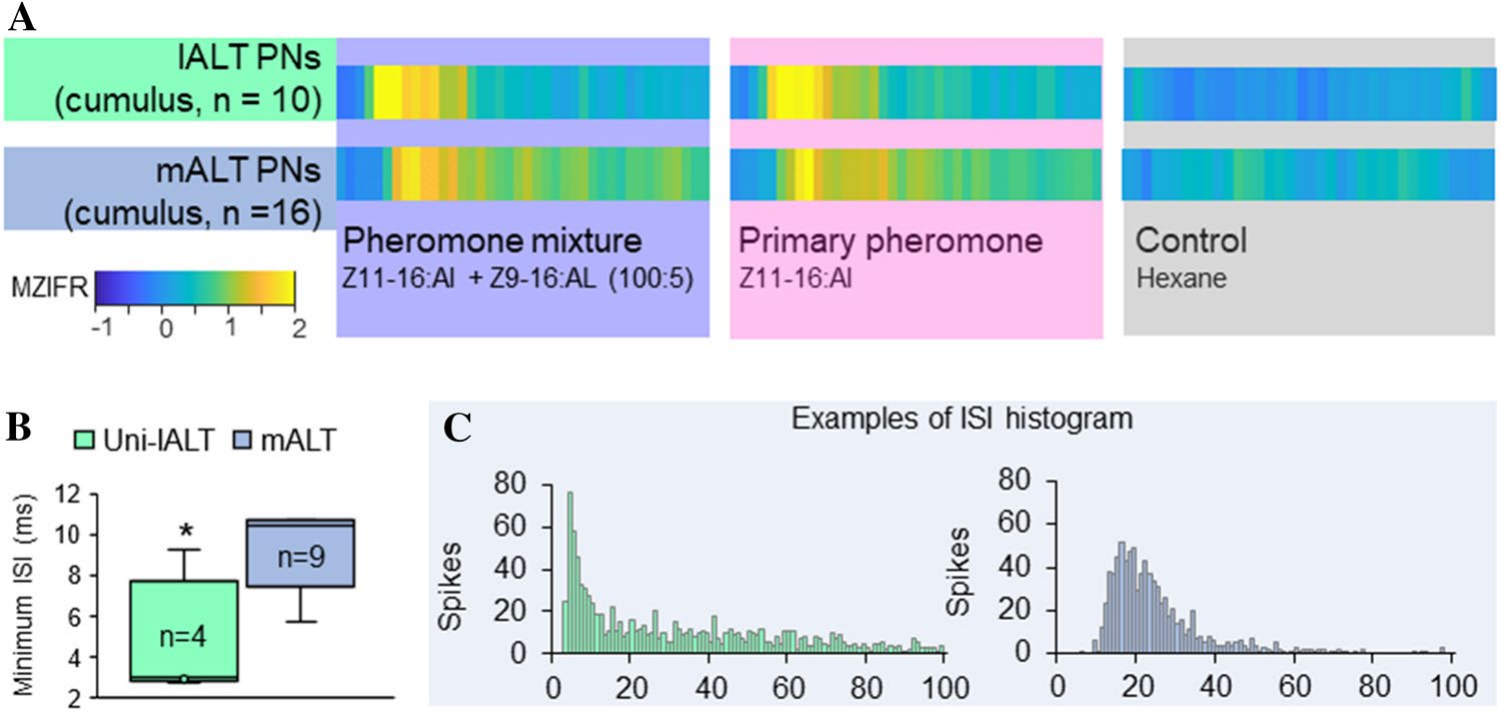
MGC PNs in the medial and lateral tract display different physiological properties. (**A**) Intracellular recordings from male-specific PNs connected with the cumulus showed that the lateral-tract PNs had a higher response amplitude and a shorter mean response delay to the pheromone stimuli than the medial-tract cumulus PNs. (**B**) The mean minimum interspike interval (ISI) was significantly shorter in the unilaterally projecting lALT PNs than in the mALT PNs. (**C**) Example histograms of ISI showed a more Poisson-like distribution in the lateral-tract cumulus PNs than medial-tract PNs. MZIFR, mean z-scored instantaneous firing rate. Data is adapted from Chu et al. (2020a) and Kymre et al. (2021b)

Physiological properties of AL output neurons are reported in different lepidopteran species—mainly in the pheromone system. Notably, electrophysiological investigations from *H. armigera* have demonstrated that response latencies were substantially shorter in lALT MGC neurons than in corresponding mALT PNs (Fig. 4; Chu et al. 2020a; Kymre et al. 2021b). The rapid responses in the lALT PNs appear to be associated with their capability of firing successive action potentials at a fast rate—the lateral-tract MGC PNs have a significantly lower minimum interspike interval than those of the medial tract during spontaneous activity (Chu et al. 2020a). Thus, different from the medial-tract MGC PNs conveying fine-tuned odor information to distinct protocerebral regions, the lateral-tract sends rapid signals via PNs which have relatively short axons and highly condensed projection terminals. Chu et al. (2020a) hypothesized that these lALT PNs could be important for rapid control of hard-wired behavior, such as optomotor anemotaxis during odor-evoked flight (Kennedy and Marsh 1974). While the characteristic convergence of lateral-tract PN terminals within the column have been found in several flying moths, it has not been reported in *B*. *mori* (Kanzaki et al. 2003)—which is a non-flying species. Since AL mass-staining data does not always display the column, we assume that the relevant PN sub-type, named Pl_a, connects only with a subset of the glomeruli. Regarding the male-specific Pl_a neurons in *H. armigera*, all identified so far originated from the cumulus—none from the two smaller MGC-units (Chu et al. 2020a). It is also noteworthy that a significant proportion of the lALT cumulus PNs, all of which innervated the column in *H. armigera* (Chu et al. 2020a), were bilateral neurons also innervating other protocerebral regions (Lee et al. 2019; Wu et al. 1996). The bilateral projections indicate potential involvement in spatial orientation, which is facilitated by bilateral connectivity (reviewed in Dalal et al. 2020).

In addition to the lALT PNs described above, six other sub-types have been reported (Fig. 3; see Table 1). Most of them are multiglomerular and have terminal projections mainly innervating ventrally located neuropils. As this includes the posterior slope and ventrolateral protocerebrum, regions associated with descending output towards the ventral nerve cord (Hsu and Bhandawat 2016; Namiki et al. 2018; Namiki and Kanzaki 2016a), olfactory processing along the lALT pathway may contribute to particularly rapid behavioral responsivity. The ventral projection pattern of the lALT implies that overlap between the medial and the lateral tract is limited, although the minor Pl_c sub-type resembles mALT PNs as it innervates the LH and the calyces (Hansson et al. 1994; Homberg et al. 1988; Rø et al. 2007; Kymre et al. 2021a). Many of these neurons, however, appear to be particularly associated with ventroposterior glomeruli (Kymre et al. 2021a), possibly indicating a role in non-olfactory processing (discussed further below). Conversely, other lALT PNs have dendrite-like arbors spreading throughout the entire antennal lobe, implying broad roles in olfactory processing. Amongst the most remarkable neurons in this tract, the Pl_d sub-type contains oligo- or omniglomerular innervations along with thin branches innervating centers associated with mechanosensation and taste, suggesting a role in multimodal integration (Homberg et al. 1988; Kymre et al. 2021a). The potentially multimodal function of this PN sub-type, which is also found in *D. melanogaster* (see Fig. 6D in Tanaka et al. 2012a), has not yet been experimentally confirmed. Profusely branched axons of the Pl_d neurons target several ventral neuropils, which occasionally even includes the lateral accessory lobe (Kymre et al. 2021a). The last-mentioned neuropil is of particular interest due to its role in pheromone-related orientation and locomotion (Namiki and Kanzaki 2016b), and is more commonly innervated by third- or fourth-order olfactory neurons.

### The mediolateral tract

The mlALT in moths is the thinnest of the main tracts, consisting of ⁓ 120 fibers (Homberg et al. 1988). Generally, these PNs are multiglomerular and bypass the calyces. Since this tract innervates the LH heavily, like the medial-tract PNs, it seems to be closely linked with the most prominent tract. About half of the mlALT PNs utilizes the inhibitory transmitter GABA (Berg et al. 2009; Hoskins et al. 1986), indicating that third-order olfactory neurons in the LH receive convergent excitatory and inhibitory inputs from the medial and mediolateral tracts, respectively—as shown in *D. melanogaster* (Wang et al. 2014).

**Fig. 5:**
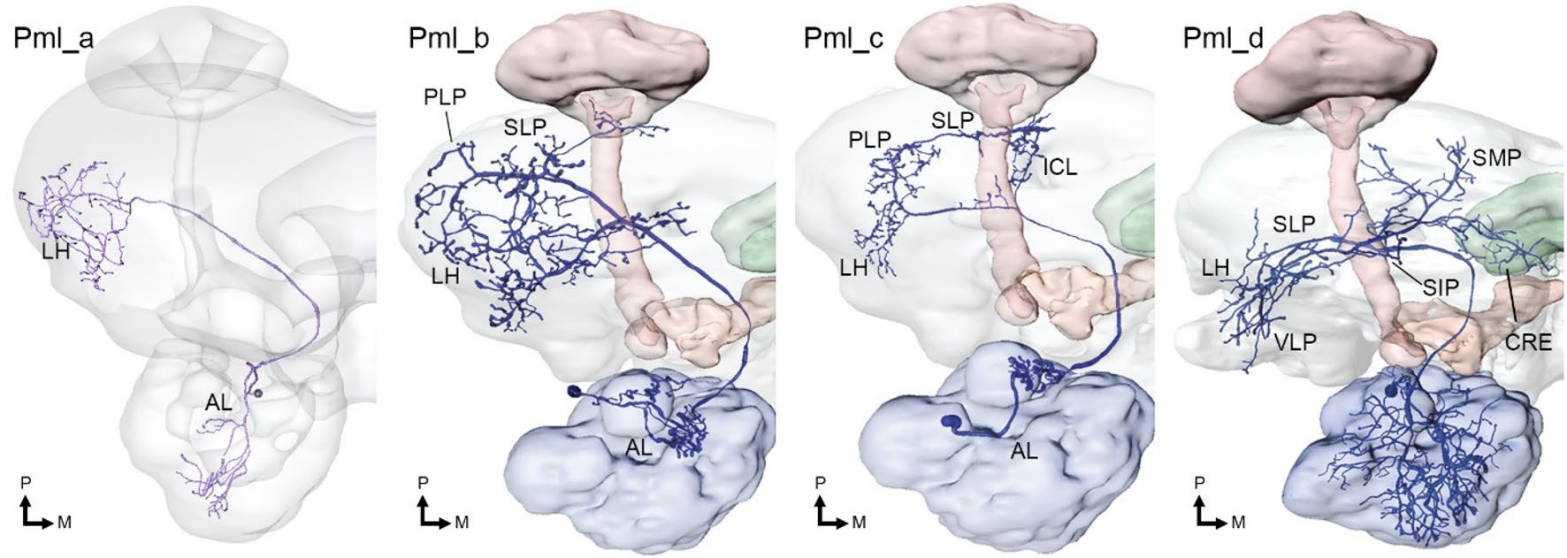
Reported mediolateral-tract PN sub-types in Lepidoptera. *AL*, antennal lobe; *CRE*, crepine; *ICL*, inferior clamp; *LH*, lateral horn; *PLP*, posteriorlateral protocerebrum; *SIP*, superior intermediate protocerebrum; *SLP*, superior lateral protocerebrum; *SMP*, superior medial protocerebrum; *VLP*, ventrolateral protocerebrum. Adapted from Ian et al. (2016b), and Kymre et al. (2021a)

So far, four mlALT PN sub-types (Fig. 5) have been identified, of which all but one tend to target both the LH and the SLP, while innervations in various other dorsally located neuropils differ between sub-types (Table 1). As such, the mlALT differs strongly from the lALT, where most sub-types target ventral brain regions. Interestingly, recent data of individually labeled mlALT PNs in *H. armigera* indicate that there is a separation of their projection terminals in the protocerebrum according to whether they are linked with the MGC or the ordinary glomeruli. Specifically, the mlALT PNs with innervation in the MGC runs to the anterior parts of the SLP and/or the ventrolateral protocerebrum (Kymre et al. 2021b), while corresponding PNs with dendrites in ordinary glomeruli generally innervate more posterior parts of the SLP as well as the LH (Kymre et al. 2021a). This pattern of functional segregation between anterior and posterior parts of the SLP is upheld also by the other antennal-lobe tracts. The comprehensive electrophysiological data from MGC PNs in *H. armigera* included findings from the mlALT-type as well (Kymre et al. 2021b). Briefly summarized, mlALT PNs with uniform innervation across the MGC glomeruli were broadly tuned to relevant pheromone components, whereas a PN with denser innervation of the cumulus than other MGC glomeruli was significantly excited only by the primary pheromone component.

Taken together, we can conlude that all the main ALTs—the medial, lateral, and mediolateral—convey signals about both plant odorants and pheromones. However, whereas the medial and the mediolateral tract share similar output areas and therefore seem to collectively contribute to common functions, such as fine-tuned odor coding, the lateral tract largely target unique output areas, indicating distinct roles, for example initiation of odor-evoked flight behavior. Roughly summed up, the two arrangements of the main antennal-lobe tracts might be compared with the ventral and dorsal stream of the mammalian visual pathway, which encode ‘what’ and ‘where’, respectively (Livingstone and Hubel 1988). Here, relevant signals from uniglomerular mALT PNs participating in odor discrimination would provide the insect with information regarding *what* its chemical surroundings include. Even though it is not immediately evident how the insect can assess *where* an odor originates from, two observations indicate that the lALT PNs play a key role in localizing an odor source: 1) the relevant Pl_a neurons carry olfactory signals to the SIP, which is located immediately adjacent to the visual anterior optic tubercle. The SIP has previously been suggested as a location for multimodal integration between visual and olfactory pathways (Lee et al. 2019), and multimodal neurons with innervations in this region were recently reported in an investigation regarding a pheromone pathway in *D. melanogaster* (Taisz et al. 2022). 2) In electrophysiological terms, the lALT PNs respond in a faster manner than mALT PNs, indicating that the contrast of signal timing between the two antennae is enhanced for lALT PNs as compared to mALT PNs. Consider a theoretical example where, in a pheromone plume, it takes pheromone molecules 10 ms to travel from the left antennae to the right one. In experimental settings, the cumulus lALT PNs have a mean response delay of 55 ms following stimulation onset, whereas the cumulus mALT PNs have an 85 ms mean response delay (Chu et al. 2020b). Thus, the contrast timing between the left and right SIP, where the lALT PNs terminate, takes on a ratio of 0.18 (10 ms/55 ms). The corresponding contrast for the mALT PNs projecting to the SLP takes on a ratio of 0.12 (10 ms/85 ms). The higher contrast between the left and right SIP, as compared to that of the left and right SLP, appear more beneficial to the quick spatial orientation required for successful pheromone tracing. In addition, the relatively shorter projection pathway of the lALT PNs as compared to that of the mALT PNs would further enhance the contrast timing across the two hemispheres.

## The three minor ALTs—the transverse, dorsomedial, and dorsal tract

Notably, while each of the three main ALTs seems to be connected with all antennal-lobe glomeruli, at least two of the three minor tracts appear to connect only with a certain proportion of glomeruli.

### The transverse ALT

**Fig. 6:**
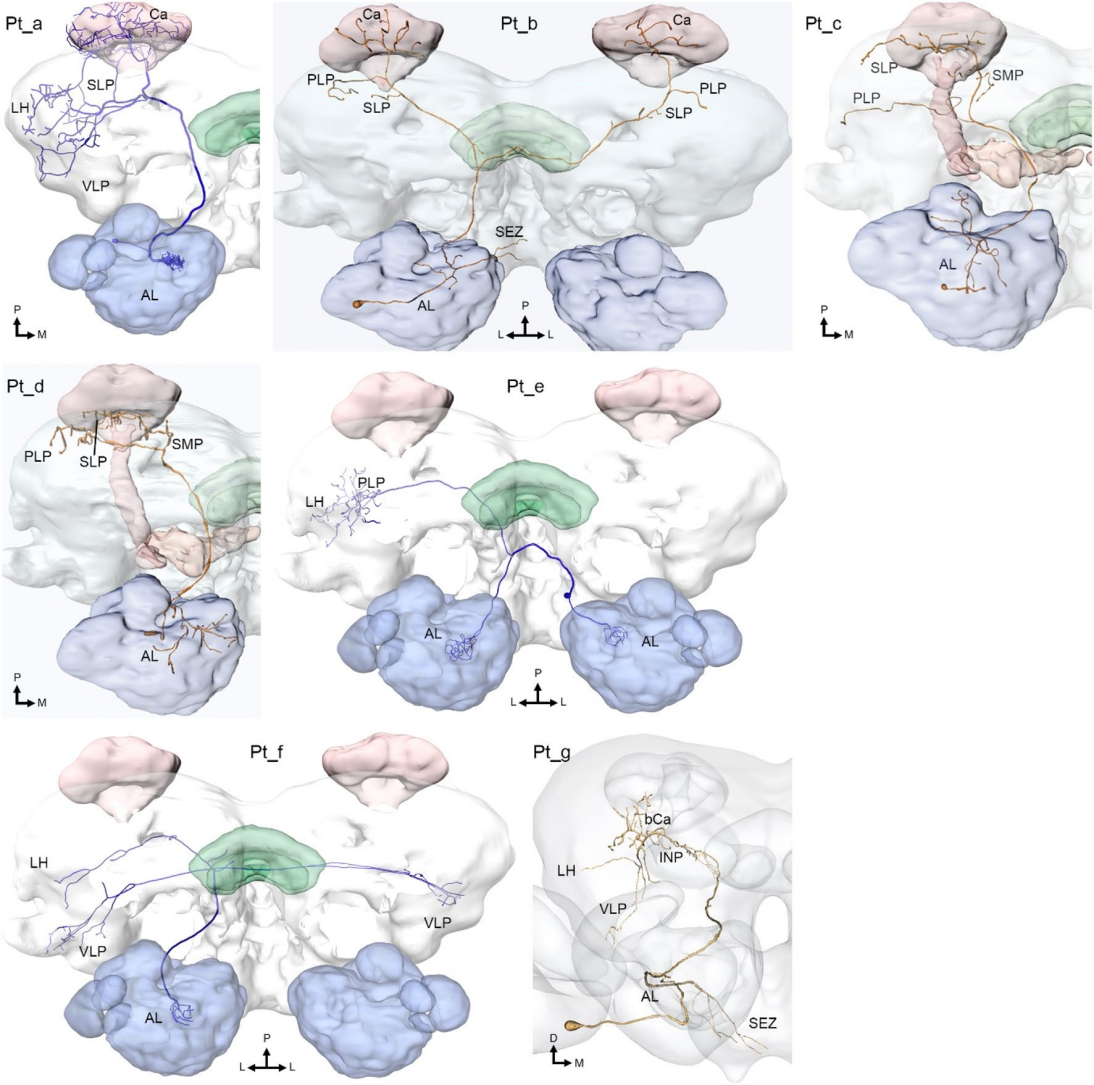
Reported transverse-tract PN sub-types in Lepidoptera. *AL*, antennal lobe; *bCa*, base of the calyces of the mushroom body; *Ca*, calyces; *INP*, inferior neuropils; *LH*, lateral horn; *PLP*, posteriorlateral protocerebrum; *SEZ*, subesophageal zone; *SLP*, superior lateral protocerebrum; *SMP*, superior medial protocerebrum; *VLP*, ventrolateral protocerebrum. Adapted from Ian et al. (2016b), Kymre et al. (2021a), and Chu et al. (2020b)

The PNs following the tALT were considered to be part of the medial tract until the tALT was identified as a unique tract—first in *D. melanogaster* (Tanaka et al. 2012b) and then in the heliothine moth (Ian et al. 2016a). In other insect orders, such as Hymenoptera and Blattodea, it is plausible that some tALT PNs have been classified as constituting one of the multiple reported mediolateral tracts (Kirschner et al. 2006; Malun et al. 1993). In heliothine moths, this relatively thin tract is formed by morphologically heterogeneous PNs originating from a limited assembly of glomeruli, often located ventroposteriorly in the AL (Chu et al. 2020b; Kymre et al. 2021a). Seven transverse-tract sub-types have been reported (see Fig. 6; Chu et al. 2020b; Ian et al. 2016b; Kymre et al. 2021a), of which five target the posteriorlateral protocerebrum (PLP), a region which is not commonly associated with olfactory processing.

Interestingly, a recent study indicated that many tALT PNs in *H. armigera* are dedicated to conveying information about the presence of environmental CO_2_. Here, it was demonstrated that the stained PNs originating from the large LPOG, located most ventrally in the AL and receiving input from the labial pit, were confined mainly to the tALT (Chu et al. 2020b). In fact, it is not unlikely that the majority of tALT PNs in the moth plays a role in conveying non-olfactory signals since most reported PNs in this tract innervate either the LPOG or the most ventroposteriorly located glomeruli. As already mentioned, similar glomeruli, and tALT PNs resembling those reported in *H. armigera*, are known to be involved in processing information about temperature and humidity in *D. melanogaster* (Enjin et al. 2016; Frank et al. 2015, 2017; Marin et al. 2020).

### The dorsomedial ALT

**Fig. 7:**
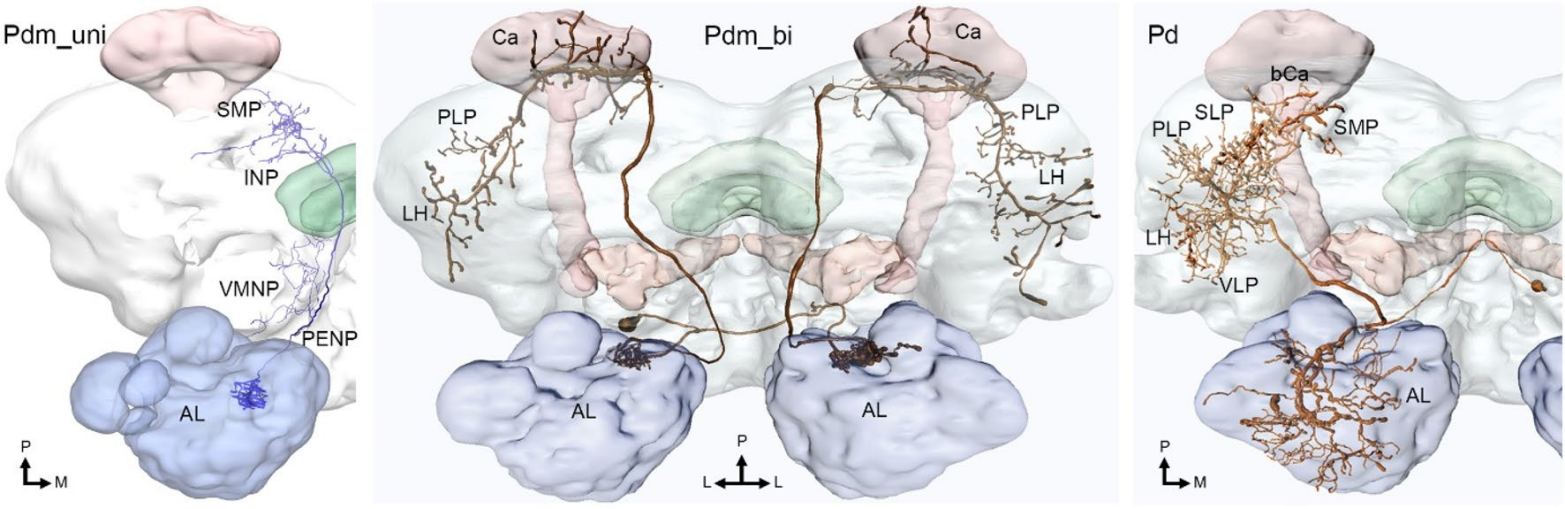
Reported dorsomedial- and dorsal-tract PN sub-types in Lepidoptera. *AL*, antennal lobe; *bCa*, base of the calyces of the mushroom body; *Ca*, calyces; *INP*, inferior neuropils; *LH*, lateral horn; *PLP*, posteriorlateral protocerebrum; *SEZ*, subesophageal zone; *SLP*, superior lateral protocerebrum; *SMP*, superior medial protocerebrum; *VLP*, ventrolateral protocerebrum. Adapted from Ian et al. (2016b), Kymre et al. (2021a), and Chu et al. (2020b)

Till now, the identified PNs passing along the dmALT (Fig. 7) are mainly bilateral neurons arborizing within one glomerulus in each antennal lobe and innervating corresponding protocerebral regions in the two hemispheres. The recent data from *H. armigera*, including four individually labeled PNs of this type, give us an accurate overview of their full morphologies. Each of the four stained PNs were connected with one of two adjacently located glomeruli. According to the glomerular map of this species (Zhao et al. 2016), two of the labeled PNs originated from glomerulus number 71 and two from glomerulus 72 (Kymre et al. 2021a). Interestingly, these two glomeruli are located most ventroposteriorly in the antennal lobe, next to the LPOG, which also happens to be innervated by a uniglomerular and unilateral dmALT PN (Chu et al. 2020b). The bilaterally projecting dmALT PNs terminated onto corresponding regions of the two hemispheres, i.e., the calyces, LH, and PLP, while their somata were positioned in the subesophageal zone. In a previous study of a corresponding PN in *B. mori*, it was reported that high concentrations of plant-odor related stimuli applied to the antenna contralateral to the cell body of the neuron induced inhibitory responses while ipsilateral stimulation had no effect (Kanzaki et al. 1989). In dmALT PNs of *H. armigera*, 11 separate odorants elicited phasic inhibition followed by a strong release-inhibition effect; however, the head space of a host plant (sunflower leaves) was associated with a prolonged inhibition (unpublished findings). Whereas the role of the bilateral dmALT PNs is not clarified in Lepidoptera, clues to their function may be found in *D. melanogaster*. There, neurons with near identical output targets, soma location, and bilateral morphologies have dendrites in the glomeruli VP1l, VP1d or VP3, which process temperature-changes or evaporative cooling (Marin et al. 2020).

### The dorsal ALT

The dALT (Fig. 7) is so far the most poorly described tract and is to our knowledge the only path with no known homologues outside the lepidopteran order. It is formed by PN axons making a sharp lateral turn immediately after leaving the AL, from where the dorsally projecting fiber bundle terminates in widespread protocerebral regions in the ipsilateral hemisphere. Remarkably, the somata of the dALT PNs are positioned close to the brain midline in the contralateral hemisphere, in the ventral part of the medial cell-body cluster of the associated AL. Whereas previous reports on *M. sexta* have found at least 30 dorsal-tract PNs (Homberg et al. 1988), repeated mass-staining experiments on heliothine moths have indicated a significantly lower number in this sub-genus, i.e., approximately 10 (unpublished data). Contrary to the findings in *M. sexta*, we found no dorsal-tract PNs distinctly connected with the MGC in *H. armigera*. Rather, it seems that the dALT in heliothine moths connects only with ordinary glomeruli (unpublished data). Generally, the dALT PNs are reported to be GABAergic (Berg et al. 2009; Hoskins et al. 1986) and they should thereby offer inhibitory signals to several protocerebral neuropils. Thus far, there are only two examples of individually labeled PNs confined to the dALT (Kanzaki et al. 1989; Kymre et al. 2021a). These Pd neurons, arborizing in many ordinary glomeruli, innervated widespread protocerebral areas in the ipsilateral hemisphere. The dALT resembles the mlALT in many respects; not only do both tracts contain many GABAergic neurons, they also have PNs with dendritic trees covering large parts of the AL and terminal projections targeting broad protocerebral areas. These tracts also project to the SLP, which is known to be targeted by other types of second-order neurons (Kymre et al. 2021a), and third-order olfactory neurons as well (Namiki and Kanzaki 2019), indicating that PNs of the mediolateral and dorsal tracts could influence other olfactory neurons at multiple levels of processing.

Altogether, the three minor tracts do not appear comparable to the main tracts—owing to their limited innervation patterns within the AL and the functional implications thereof. Two of the minor tracts, the transverse and dorsomedial ALTs, are specialized to processing signals associated with a select group of glomeruli, which includes the LPOG, VPGs, and a few ordinary glomeruli. While the dorsal tract has quite broad innervations within the AL, it has not appeared in mass stains from the MGC (Chu et al. 2020b), nor from the LPOG (Chu et al. 2020a), indicating that dALT PNs are not involved in processing input about pheromones or CO_2_.

## PN outputs are spatially stereotyped according to dendritic innervation patterns

**Fig. 8:**
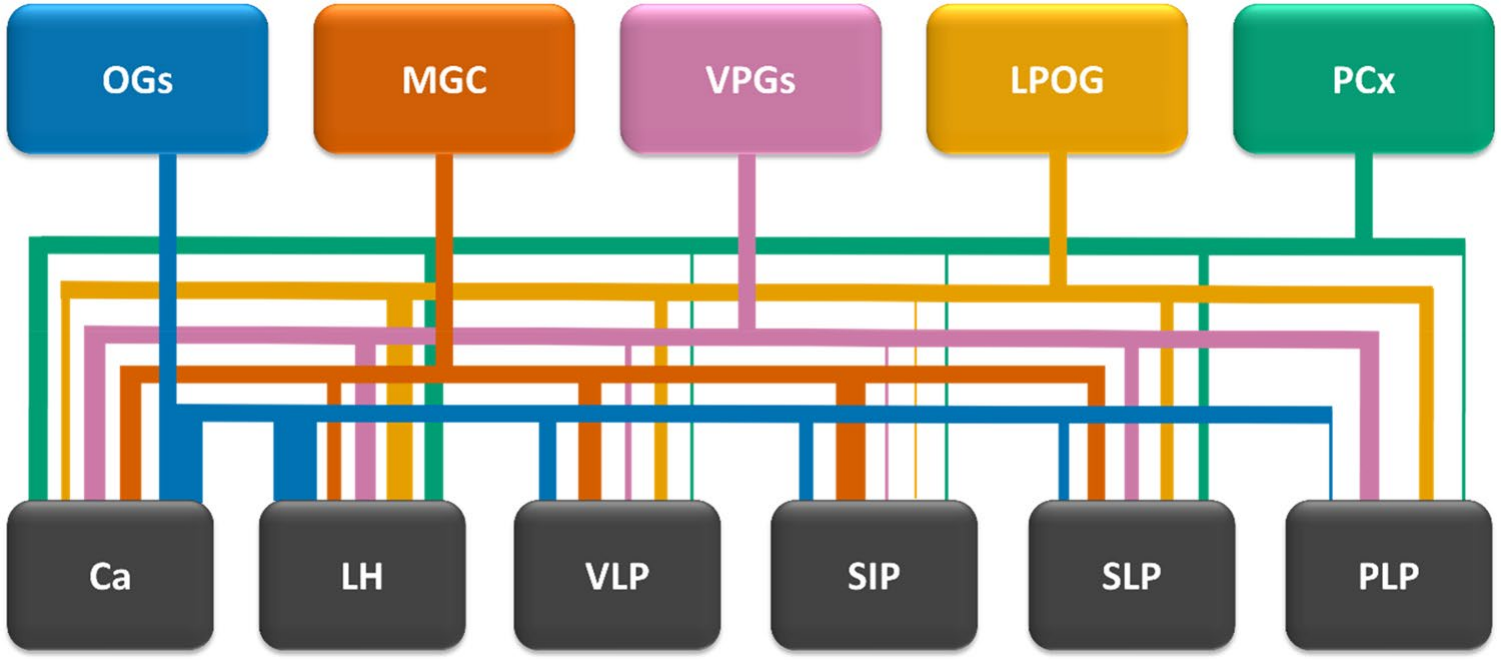
Schematic overview representing approximate proportions of output from distinct glomerular clusters. The thickness of the colored bars indicates the proportions of outputs from each specific glomerular cluster onto distinct protocerebral neuropils, note that this thickness is not comparable across the glomerular clusters. Moreover, this representation does not extend to the total amount of inputs each higher-order olfactory region receives across glomerular clusters. The scheme was created based on visual inspections of individual neuron labeling and selective mass staining from separate antennal-lobe sub-systems (Chu et al., 2020a, b; Kymre et al., 2021a, b). *Ca*, calyces of the mushroom body; *LH*, lateral horn; *LPOG*, labial palp-pit organ glomerulus; *MGC*, macroglomerular complex; *OGs*, ordinary glomeruli; *PCx*, posterior complex; *PLP*, posteriorlateral protocerebrum; *SIP*, superior intermediate protocerebrum; *SLP*, superior lateral protocerebrum; *VLP*, ventrolateral protocerebrum; *VPGs*, ventroposterior glomeruli

The large number of newly characterized PNs in recent years, based on high-resolution confocal images of individually labeled neurons, has also allowed for new insights into the connections between separate AL sub-systems and protocerebral neuropils (summarized in Fig. 8). PN output is spatially stereotyped according to behaviorally relevant features in the lateral protocerebrum of various insect species (Jefferis et al. 2014; Seki et al. 2005; Zhao et al. 2014), the mammalian amygdala (Sosulski et al. 2011), and the habenula in fish (Miyasaka et al. 2014).

### Lateral horn

As has been reported for all investigated moth species (see Table 1), the LH is undoubtedly the main target of AL PNs, although it does not represent all functional AL sub-systems equally. The LH receives substantial inputs from the OGs, VPGs, PCx (Kymre et al. 2021a), and LPOG (Chu et al. 2020b), whereas connections to the MGC mainly include the small glomeruli tuned to minor pheromones, and not the cumulus receiving the uniquely attractive main pheromone signal (Chu et al. 2020a; Kymre et al. 2021b). In addition, reports from *Helicoverpa assulta* (Zhao et al. 2014) and *B. mori* (Seki et al. 2005) have found that the most lateral innervations of MGC PNs are situated anteriorly to the terminals associated with OGs. Such a functional segregation within the LH has also been reported in *D. melanogaster* (Jefferis et al. 2007).

### Superior neuropils

The SLP is another lateral-protocerebral region of particular interest to olfactory processing, as it receives varying extent of input from five out of six tracts (see Table 1). In addition, this neuropil is the main output area of LH output neurons (Namiki and Kanzaki 2019). Altogether, this suggests that neurons with dendrites in the SLP may receive combined inputs from multiple orders of olfactory processing. Second-order PN outputs onto the SLP are segregated according to dendritic arborization—the anterior part of the SLP is targeted by PNs innervating the cumulus of the MGC, which represents attraction-related signals from conspecific females (Kymre et al., 2021b), whereas the posterior SLP receives terminals from PNs representing glomeruli associated with plant odorants, CO_2_, and potentially temperature and humidity (Chu et al. 2020b; Kymre et al. 2021a). The column of the adjacent SIP, which is located medial to the most anterior parts of the SLP, receives converging projection terminals originating from the MGC and the ordinary glomeruli (Chu et al. 2020a). The remaining AL sub-systems, i.e., the LPOG, VPGs, and PCx, appear to only be connected with the SIP via PNs with dendrites in most or all glomeruli (e.g., mlALT PNs, Kymre et al. 2021a), the functional specificity of these multiglomerular PNs remains unknown.

### Ventrolateral neuropils

Although often overlooked, the VLP is a neuropil which receives inputs from highly divergent PN sub-types, including a particularly large number of PNs in the lALT. However, the VLP receives inputs from all AL sub-systems via several tracts and is the only brain region being innervated by uniglomerular mALT PNs originating from all three MGC glomeruli (Kymre et al. 2021b)—similar to the lateral part of the delta region in *B. mori* (Seki et al. 2005). As for the adjoining PLP, it seems that this region may be particularly relevant with respect to non-olfactory signaling pathways originating in the AL. Indeed, both the VPGs and LPOG seem to be heavily represented in this part of the lepidopteran brain (Chu et al. 2020b; Kymre et al. 2021a). Notably, reports from the fruit-fly link this region with thermo- and hygro-sensory AL output (Frank et al. 2015, 2017; Liu et al. 2015).

### Mushroom body calyces

Finally, the calyces are of course a major target of PNs confined to the mALT. In addition, some of the tALT PNs and one of the lALT PN sub-types innervate this neuropil (Table 1). Previous studies have described calycal innervation patterns formed by PNs of different AL sub-systems—inputs from the PNs originating from OGs are widely distributed throughout the calyces, whereas MGC PNs innervate a more confined region within the inner parts of the calycal cups (Homberg et al. 1988; Zhao et al. 2014). Namiki et al. (2013) found that minor-pheromone processing PNs in *B. mori* project to areas overlapping with the terminals of PNs associated with plant odors, implying integration of inputs from these cues. The PNs associated with the main pheromone, however, were found to innervate a unique region separated from inputs regarding minor pheromones and plant odors. Other AL sub-systems are also connected with the calyces, but the terminals from the LPOG PNs in the transverse tract (Chu et al. 2020a), PCx PNs in the medial tract, as well as VPG PNs in the lateral, transverse and dorsomedial tracts (Kymre et al. 2021a) do not appear to be biased toward innervation of specific calycal regions.

## Higher-order olfactory processing

Having already mapped the AL PNs comprehensively in the heliothine moth, it would now be relevant to investigate the downstream targets of these neurons. This work has already started in several moth species, such as *M. sexta* (Kanzaki et al. 1991; Lei et al. 2013), *B. mori* (Namiki et al. 2013, 2014; Namiki and Kanzaki 2019), *Agrotis segetum* (Lei et al. 2001), and *Heliothis virescens* (Løfaldli et al. 2012). In *B. mori*, the Kanzaki group has even managed to characterize the pathway dedicated to main pheromone processing from the 2nd to 4th order neurons (Namiki et al. 2014). Higher-order processing of other signal categories, connected with AL sub-systems outside the MGC has, however, received limited attention. The study of plant-odor representation in the protocerebrum has often been restricted to stimulation with a single plant odorant. A notable exception is the work of Løfaldli et al. (2012), who included many odorants, both as single components and mixtures. As for other cues with relevance to AL PNs, such as CO_2_ and potentially temperature and humidity, no studies have thus far included such stimuli in recordings from protocerebral neuropils in Lepidoptera.

As a considerable amount of information regarding the pathways connecting separate AL sub-systems with distinct protocerebral neuropils is now available, it is possible to design future experiments aimed at studying protocerebral processing of specific AL-related stimuli. Furthermore, previous knowledge on other sensory modalities, both in Lepidoptera and other insect species, may also be utilized to investigate multimodal integration in appropriate loci. Candidate regions include the VLP, a region often associated with visual processing (Ito et al. 2014). This area also receives terminals from various types of AL PNs—some with additional dendrites in the mechanosensory and gustatory centers (Homberg et al. 1988; Kymre et al. 2021a), along with outputs from auditory and gustatory interneurons (Kvello et al. 2009; Pfuhl et al. 2014). Such a region could include a plethora of neurons integrating different combinations of sensory modalities, which have yet to be studied. The anterior SLP, along with the SIP, could also be relevant for investigations regarding putative integration of signals relating to the major sex pheromone (Kymre et al. 2021b) and visual cues—these protocerebral neuropils are proximal to the visual neuropil, the anterior optic tubercle, and is indicated in multimodal processing in *D. melanogaster* (Taisz et al. 2022). As the insect must integrate input across multiple sensory modalities to successfully navigate its surroundings, studies of multimodal integration are a prerequisite to fully understand the neuronal underpinnings of relevant behaviors, including olfaction-based behaviors. In recent years, multimodal integration has received considerable attention in several insect orders (reviewed by Buehlmann et al. 2020; Thiagarajan and Sachse 2022), although much remains to be learned in terms of how various odorants are integrated with other modalities, and which neurons partake in these computations.
